# Changes in sexual behaviour leading to the decline in the prevalence of HIV in Uganda: confirmation from multiple sources of evidence

**DOI:** 10.1136/sti.2008.029892

**Published:** 2008-09-16

**Authors:** D Kirby

## Abstract

**Objectives:**

To identify the changes in sexual behaviour that led to the dramatic reduction in the prevalence of HIV in Uganda in the early 1990s.

**Methods:**

Seven different types of evidence were examined: (1) models of HIV prevalence and incidence in Kampala and other sentinel sites in Uganda; (2) reports of behaviour change in the primary newspaper in Uganda; (3) surveys with questions about perceptions of personal behaviour change; (4) large demographic and health surveys (DHS) collected in 1988/9 and 1995 and large Global Program on AIDS (GPA) surveys in 1989 and 1995 with questions about reported sexual behaviour; (5) smaller less representative surveys of reported sexual behaviour collected in other years; (6) reports of numbers of condoms shipped to Uganda; and (7) historical documents describing the implementation of HIV prevention programmes in Uganda.

**Results:**

All seven types of data produced consistent evidence that people in Uganda first reduced their number of sexual partners prior to or outside of long-term marital or cohabiting relationships, and then increased their use of condoms with non-marital and non-cohabiting partners.

**Conclusions:**

Consistent with basic theories about transmission of sexually transmitted infections, first reducing the number of sexual partners and breaking up sexual networks and then reducing the chances of HIV transmission with remaining casual partners by using condoms can be achieved and can dramatically reduce the sexual transmission of HIV in generalised epidemics.

Despite decades of effort, the prevalence of HIV is still very high in some countries, especially those with generalised epidemics in sub-Saharan Africa.^1^ Unfortunately, trials of vaccines to inoculate people against HIV have not had success,^2^ and some of the most promising approaches to reducing HIV transmission continue to involve behaviour change.^3^ ^4^ However, consensus does not exist on which, if any, sexual behaviour(s) can be changed and can in turn dramatically reduce HIV transmission in generalised epidemics in sub-Saharan Africa. In fact, there is considerable disagreement among those professionals favouring the promotion of abstinence, condom use, being faithful or other risk reduction behaviours.^3^ ^5^ ^6^ Views on these issues have a large impact on the levels of funding and other resources that are devoted to promoting each risk reduction behaviour.

One of the most dramatic reductions in the prevalence of HIV occurred in Uganda in the early 1990s.^7^ ^8^ Part of the explanation for the decline in prevalence can be attributed to factors involved in the natural course of an epidemic. In many epidemics those people who are most vulnerable are infected first and, after they have become infected, the incidence declines. Other factors such as improvements in the blood supply also contributed to the reduction of HIV in Uganda. Nevertheless, researchers have concluded that the most important factors were changes in sexual behaviour.^4^ ^8^^–^11

Despite this overall conclusion, differing views and questions persist regarding *which* sexual behaviours changed, *when* they changed and which made the greatest contribution to the reduction in HIV incidence.^10^ ^12^^–^19 Answering these questions about Uganda can help inform the development and implementation of effective programmes in other sub-Saharan countries with generalised epidemics.

Rather than present the evidence from one scientific method, this paper takes a different approach to help answer these questions; it reviews seven kinds of evidence and examines the consistency among them. More specifically, it examines: (1) models of HIV prevalence and incidence in Kampala and other sentinel sites in Uganda; (2) articles about sexual behaviour change in the primary newspaper in Uganda; (3) surveys with questions about perceptions of personal behaviour change; (4) large demographic and health surveys with questions about reported sexual behaviour; (5) smaller less representative surveys of reported sexual behaviour; (6) written reports of numbers of condoms shipped to Uganda; and (7) historical documents describing the implementation of HIV prevention programmes in Uganda. Because of space limitations, it is not possible to discuss the strengths and limitations of each type of evidence. Rather, the strength of the conclusions of this paper lies in the consistency of the findings across the different sources of evidence.

## MODELLING OF INCIDENCE AND PREVALENCE RATES OF HIV

Official HIV prevalence rates are available for Kampala (the capital), four major towns and 14 rural areas.^7^ The earliest data available for Kampala are from 1985. The sites were clinics or hospitals located in all but the northern parts of Uganda that used blood tests to determine the prevalence of HIV in women obtaining antenatal care. Despite limitations (eg, some small sample sizes, missing data for some years and reliance on women receiving antenatal care), these estimates of HIV prevalence have generally been confirmed by estimates from other sources in Uganda (eg, blood tests among women seeking voluntary HIV tests at AIDS information centres in Kampala for the first time)^7^ ^20^ and provide the best estimates of how prevalence changed over time in different parts of Uganda during the late 1980s and 1990s.

**Figure 1:**
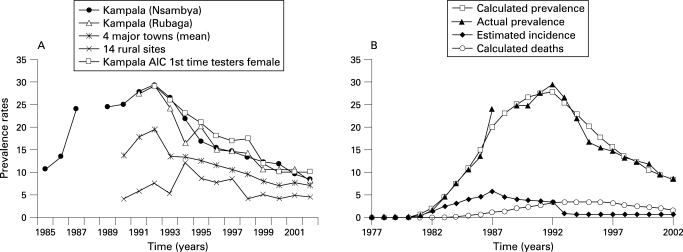
(A) Prevalence of HIV in Uganda. (B) Estimated incidence, prevalence and death from HIV in Kampala (Nsambya Hospital) assuming a 1987 peak and period of 8 years.

Prevalence data for Kampala indicate that the prevalence increased very rapidly through 1987, increased at a slower rate in 1988 until it peaked in 1992, decreased rapidly in 1993 for several years and continued to decline through 2002 (fig 1A). In the four major towns prevalence also increased, on average, through 1992 and then, as in Kampala, began a rapid decline in 1993 and continued to decline through 2000. In the rural areas the pattern is less clear because the trend line is based on different sites and because prevalence began to decline in different sites at different times. However, in many rural sentinel sites, prevalence began to decline between 1992 and 1997.

Because of the time lag between decreases in incidence and decreases in prevalence during the early stages of many HIV epidemics, most models of decreases of incidence in Kampala indicate that there was roughly a 5-year time lag between the decrease in incidence and the decrease in prevalence. Thus, nearly all plausible models consistent with the prevalence data indicate that the incidence of HIV in Kampala and the major towns peaked between 1986 and 1989 and then began a continuous decline for several years.^15^

However, the model of incidence that best fits the prevalence data indicates that the incidence of HIV in Kampala increased rapidly through 1987, peaked in 1987 or 1988, declined gradually between 1988 and 1992 and declined more rapidly in 1993 (fig 1B).^21^ These data suggest that there may have been two points in time when incidence changed the most: one in 1987 or 1988 when incidence peaked and then began declining, and a second in 1992/3 when incidence dropped more rapidly.

Because prevalence data for the four major towns are not available before 1990, it is much less clear when incidence peaked in those towns. However, plausible modelling indicate that, in the four major towns, incidence peaked between 1989 and 1991, began to decline by 1992, and then declined dramatically in 1993 and remained low.^21^

Unfortunately, the prevalence data for most rural sentinel sites are not sufficiently complete—especially in the earlier years—to attempt to model changes in incidence, and thus it is difficult to determine exactly when incidence began to decline in most of them.

In sum, modelling of incidence suggests that the incidence probably peaked about 1987/8 in Kampala and declined more rapidly in 1993, and in four major towns it probably peaked between 1989 and 1991 and then declined rapidly. All of this suggests that sexual risk behaviour may have begun to change significantly around 1987 or before.

## NEWSPAPER REPORTS OF BEHAVIOUR CHANGE

Given that large surveys of sexual behaviour were not conducted in Uganda until 1988/9, newspaper accounts may provide the earliest documentation of behaviour change, even though these accounts are not necessarily representative. Uganda’s primary newspaper in English is *The New Vision*.

*The New Vision* first mentioned behaviour change in response to AIDS in a January 1987 article about a fishing village in Rakai hard hit by deaths from AIDS.^22^

[Men]…visit the town’s 200 or so prostitutes less often now… But since the smuggling has stopped and since people learnt that AIDS is transmitted sexually, business has become poor, and about 100 prostitutes have returned to their villages in Tanzania. “Before it was impossible to stay in a lodge without a woman,” said one man. “They would book all the rooms and then look for a man. Now there are fewer women and you can sleep alone if you want.” Many men now use condoms, which are available in the village and cost only 500 shillings.

*The New Vision* first mentioned behaviour change in Kampala resulting from AIDS in August 1987:

It is not so good for the prostitutes. “Slim” (AIDS) has ruined business. Irrespective of the fact that for as little as twenty (20) shillings, one can get service, few customers are turning up except perhaps when drunk.^23^

In the autumn of 1987 *The New Vision* devoted two-thirds of an entire page to the topic of how HIV had changed behaviour. The article described how “naïve complacency” was replaced by real concern caused by seeing relatives and friends die of AIDS. As a result, “the horror of Slim is forcing people to change social habits”. The article described the rewards to wives and also the challenges to men of staying home more and attempting “zero grazing”. It described how quick sexual liaisons with barmaids were less frequent and how “the threat of Slim is forcing a large cross-section of people to change their ways”.^24^

Two months later *The New Vision* briefly described how fear of AIDS was causing “loose men and women” to cease attending cinemas, discos, restaurants and hotels in another part of the country.^25^

In these 1987 articles there were only brief mentions of condom use and only one article about condom use in Kampala. That article indicated that condom use was increasing in Kampala, but the only evidence it provided was the “sudden profusion of discarded condoms”.^24^

By the summer and autumn of 1989 there were a few additional newspaper articles stating that sexual behaviour had begun to change. According to these articles, it was observed that people were changing their sexual behaviour and that zero-grazing had become fashionable as a result of AIDS.^26^ Thereafter, newspaper accounts often presented the results of surveys, discussed below.

In sum, these newspaper articles are consistent with the modelling of HIV prevalence and incidence, and suggest that sexual behaviour had begun to change in Kampala and some other parts of Uganda by the end of 1987. These reports also indicated that people were reducing their sexual risk primarily by having less casual sex in these early years and secondarily by using condoms.

While newspaper articles can accurately date events, they cannot provide strong evidence for the magnitude of changes in sexual behaviour nationwide. Only representative surveys can provide such evidence.

## SURVEY QUESTIONS ABOUT PERCEPTIONS OF PERSONAL BEHAVIOUR CHANGE

By far the two largest and most representative surveys in Uganda were the Demographic and Health Surveys (DHS) and the World Health Organization’s Global Program on AIDS (GPA) survey.^27^ Both the GPA survey conducted in 1989 and the DHS survey conducted in 1995 asked respondents if and how their behaviour had changed as a result of HIV/AIDS. The results should be viewed cautiously, since some respondents may have claimed they had changed their behaviour in socially expected ways even if they had not.

According to the 1989 GPA survey,^27^ about 30% of the respondents had made no changes in their sexual behaviour and did not intend to, 9% had already changed their sexual behaviour (avoided sex, avoided prostitutes, avoided casual sex, maintained faithful relationships) or intended to do so, 1% had begun to use condoms or intended to do so, and 60% had made or intended to make other changes (were more careful in general, avoided people with AIDS, were careful with blood, were careful with injections, etc). These data suggest that a small percentage of the population had already changed their sexual behaviour or intended to by 1989, but that this percentage was still quite small (about 10%). Furthermore, of those who had changed their sexual behaviour or intended to do so, the vast majority did so or intended to do so by avoiding casual sex and only a few used or intended to use condoms to prevent AIDS.

In 1995 the DHS survey asked respondents how their behaviour had already changed as a result of HIV/AIDS. Fifty-four percent of never-married men responded that they delayed their initiation of sex, stopped having sex, or restricted themselves to one sexual partner; only 17% said they started using condoms. Among never-married women, 67% said they delayed initiation of sex, stopped having sex, or restricted themselves to one partner; only 3% said they began to use condoms. Among married men and women, even larger percentages said they restricted themselves to one sexual partner and even fewer said they began using condoms. Other studies found similar results.^28^^–^30

Although questions about perceptions of personal behavioural change can provide estimates of the amount of behavioural change by the time of the survey, more valid estimates can be derived by examining trend data from multiple representative surveys administered at different times.

## DHS AND GPA SURVEYS OF REPORTED SEXUAL BEHAVIOUR IN 1988/9 AND 1995

The DHS mentioned above were conducted in 1988/9 and 1995. The 1988/9 sample included 4370 women but no men, while the 1995 sample included 7070 women and 1996 men, all aged 15–54 years.^31^ ^32^

The GPA surveys mentioned above were conducted in September to December 1989 and in 1995. The 1989 survey included 1443 women and 1661 men from eight different health districts, while the 1995 survey included 3089 women and 2339 men from only four of the eight districts.

The great advantage of the DHS and GPA surveys is that they asked large and either nationally or subnationally representative samples about their sexual behaviour during important years of the epidemic. In addition, their survey instruments were carefully developed and professionally administered.

In terms of understanding the changes over time in sexual behaviour that may have affected the course of the epidemic, a weakness of both surveys is that the earliest ones were not conducted until 1988/9, after people already were aware of HIV/AIDS. As discussed above, some people (roughly 10%) reported that they already had changed their sexual behaviour before the first DHS or GPA surveys.^27^ ^28^ A comparison of the 1988/9 and 1995 surveys may therefore underestimate the amount of behaviour change that took place.

The two GPA surveys had additional limitations. Although large, they were subnational, had a strong urban bias and the 1995 survey sampled only four of the eight districts sampled in 1989.

### Abstaining from sex

According to the DHS surveys, between 1988/9 and 1995 the number of women aged 15–54 years, regardless of marital status, who had sex during the previous year decreased slightly from 82% to 75%.^32^ This small decrease reflected the fact that most Ugandan women marry at an early age and have sex after marriage. Comparable data were not available for men. Among never-married women there were much larger changes, especially in urban areas. The percentage of never-married women aged 15–24 years who had sex in the previous 12 months decreased significantly from 36% in 1988/9 to 22% in 1995.^32^

According to the GPA surveys, between 1989 and 1995 the number of men aged 15–54 years, regardless of marital status, who had sex during the previous year decreased slightly from 85% to 77%.^32^

### Extramarital sex

GPA survey data indicate that the percentage of women engaging in extramarital sex was always quite low (about 6% or less) and remained stable during this period. For men, however, the GPA data suggest that there were declines in extramarital sex from 23% in 1989 to 16% in 1995.

### Sex with casual and multiple non-marital non-cohabiting partners

Only the GPA surveys included questions about sex outside marriage or about multiple partners in 1989 and 1995, and these results should be viewed somewhat cautiously because of changes in the sample and questionnaire design. According to the GPA data, between 1989 and 1995 the percentage of women (both married and unmarried) who had sex with a non-marital/non-cohabiting partner in the last 12 months decreased from 23% to 9%. Among men the percentage decreased from 41% to 21%. Similarly, between 1989 and 1995 the percentage of all women who had one or more casual partners in the last year decreased from 16% to 6% while the percentage of men who had one or more casual partners decreased from 35% to 15%. In addition, both *single* women and *single* men became much less likely to have sex with two or more sexual partners during the previous year (from 22% to 17% among single women and from 54% to 33% among single men).

In sum, all women and men regardless of marital status became a little less likely to have sex at all during the previous year, became less likely to have sex outside marital or co-habiting relationships and became less likely to have one or more casual sexual relationships. Married women maintained low levels of extramarital sex, while married men became less likely to have extramarital sex. Both single men and single women became less likely to have sex during the previous year and less likely to have sex with multiple sexual partners.

### Condom use

According to the DHS data, the percentage of sexually experienced women who had ever used a condom was very low (1%) in 1989. By 1995 it had increased to only 6%. Among men in 1995 it increased to only 16% from a low but unknown percentage in 1988/9 (men were not sampled in 1988/9). According to GPA data the percentage of women who used a condom the last time they had sex increased from 7% to 20% between 1989 and 1995, while among men it increased from 15% to 30%.

These results may give misleading impressions about the possible impact of condoms because most adults were married and most married couples rarely used condoms, if at all. More relevant to HIV transmission at that time is the use of condoms with casual partners outside of marital/cohabiting relationships. According to DHS data, the percentage of women throughout Uganda who used a condom the last time they had sex with a non-marital/non-cohabiting partner increased to 20% in 1995; among men, it increased to 36%. While these increases may have been too low to markedly reduce the incidence of HIV, increases in reported condom use were greater among men, people in urban areas, young adults and people with more education. For example, in urban areas in 1995, 62% of men used a condom the last time they had sex with a casual partner. These higher rates may have had a greater impact.

The GPA surveys conducted in 1989 and 1995 also demonstrated a large increase in condom use with non-marital/non-cohabiting partners. This increase was reported by both men and women.

## OTHER SURVEYS OF SEXUAL BEHAVIOUR

This review found only one study during this period that tracked the same cohort of people over time and measured their sexual behaviour at different points in time.^29^ That study surveyed 1204 respondents in 1987 and 1992 from a semi-rural community near Kampala. It found that the percentage of men who reported two or more partners during the previous 6 months fell from 43% to 12% and the percentage of women who reported two or more partners fell from 13% to 1%.

In addition to this cohort study, Barton reviewed 63 independently conducted cross-sectional studies between 1987 and 1996.^33^ Despite the great variation in samples, Barton concluded that “the most important changes are reduction in number of sexual partners (especially non-regular/casual partners), adoption of condom use (especially with non-regular partners), and delaying the onset of sexual activity in adolescents”. Eleven of these 63 studies collected data from the same target group at multiple times (open cohorts). In all 11 studies condom use increased, either among the entire sample (10 studies) or among the subsample having casual sex during the past year (one study).

Another survey—a non-representative survey of men in Kampala—indicated that, as early as 1993, 55% of men in Kampala used condoms with non-marital/non-cohabiting partners the last time they had sex.^34^ Thus, other surveys support the results from the DHS and GPA surveys, indicating that there were reductions in numbers of sexual partners and increases in condom use.

These quantitative studies of sexual behaviour are supported by qualitative studies of interviews with key informants and focus groups with community members. In one study key informants and focus group participants remembered that, after learning that AIDS was sexually transmitted, people stopped having sex with as many partners outside of marriage or long-term partnerships.^21^ According to many informants this was the primary behaviour change that took place in the late 1980s or early 1990s. Respondents also mentioned that, before condoms became widely available, many people lacked information about them, there was widespread fear about using them, and they were costly and rarely used. However, after condoms were promoted through social marketing campaigns, many myths were corrected and condoms became available (which varied with the remoteness of the community) and people began using them much more frequently. Respondents strongly emphasised that, in the late 1990s, after the condom social marketing campaigns had been in place, the social norm for many young people was to use condoms to avoid contracting HIV.

## REPORTS OF CONDOM SHIPMENTS TO UGANDA

People cannot use condoms if they are not available. Because Uganda did not produce its own condoms during the 1990s, the number of condoms shipped to Uganda provided an upper limit on the number of condoms that could be used.

Some studies have provided estimates of the numbers of condoms distributed through the largest social marketing campaigns^8^ ^11^ and concluded that too few condoms were distributed in Uganda to have significantly reduced the incidence of HIV. However, these estimates of condoms distributed in Uganda substantially underestimate the number that were available because they do not include those condoms that were distributed through other efforts.

For this review, many global donors who supported purchases for condoms and other contraceptives were identified and their reports on the numbers of condoms sent to Uganda were reviewed. Even this tally does not include all global donors (eg, the Swedish International Development Cooperating Agency or the European Union) or donors not operating at the global level (eg, private sector donors and those providing condoms for special marketing projects). Thus, the numbers are still underestimates.

**Figure 2:**
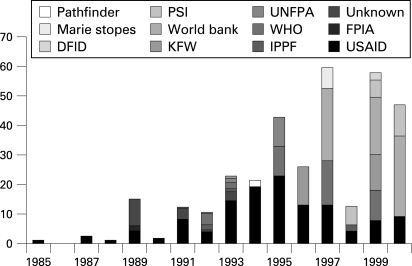
Number of condoms (in millions) received in Uganda from different organisations by year. DFID, Department for International Development; FPIA, Family Planning International Association; IPPF, International Planned Parenthood Federation; KFW, Kreditanstalt für Wiederaufbau/MBZ; PSI, Population Services International; UNFPA, United Nations Population Fund; USAID, United States Agency for International Development; WHO, World Health Organization.

According to these data, relatively few condoms were received in Uganda before 1989 (fig 2). In 1989 about 15 million condoms were received, in 1991 and 1992 about 12 million, and in nearly every subsequent year the number exceeded 20 million and grew rapidly.

While the numbers of condoms received in the early 1990s were clearly not sufficient to provide protection against HIV for all sexually active people in Uganda (which had a total population of about 17 million in 1990), there were enough condoms, especially by 1993, in Kampala and the major towns to provide protection for a substantial proportion of the acts of sex among those having sex outside marriage or a long-term relationship. These data are consistent with the 1995 DHS data showing that, in urban areas, about 62% of men who had sex with non-marital non-cohabiting partners used a condom the last time they had sex.

## DOCUMENTS DESCRIBING THE PROGRAMMATIC RESPONSES TO AIDS

Over 80 documents from the Uganda AIDS programmes and from investigators conducting research in Uganda were reviewed to examine the development of the Uganda AIDS programmes. They confirm that, in Uganda, AIDS efforts focused primarily on prevention.^4^ ^35^ While steps were taken to ensure a safe blood supply and improve clinical practices, most of the prevention efforts relied on health education strategies to reduce sexual risk.^31^ ^36^

Beginning in 1986, President Museveni, a charismatic leader, personally addressed AIDS. He strongly encouraged government and civil society to tackle AIDS. He appointed motivated and competent people to prevent the spread of AIDS and he delegated authority to them. In addition, he spoke forcefully about AIDS to the public.

The government thus initiated many efforts to stop AIDS. Over time these matured into a multisectoral approach to fight AIDS with systematic efforts involving nearly every group with an infrastructure: district health teams, the media, schools, faith communities, local councils, youth organisations, women’s groups, non-governmental organisations, prisons and other groups including traditional healers. The AIDS Control Program created a training structure including trainers and trainers of trainers so that all groups would be trained. It also began printing huge numbers of training manuals, pamphlets, posters and other educational materials.

Nearly all the organisations involved in AIDS prevention focused on clear and consistent messages. Especially during the earlier years, the messages were “be faithful”, “zero grazing” and “love carefully”. These typically translated into: If you are married or in a long-term relationship, be faithful and do not have sex with others. If you are single, wait until marriage or have only one partner. If you must have sex outside marriage or a long-term relationship, then always use a condom. In schools there was a greater emphasis on abstaining from sex.

Although some condoms were available in Uganda for family planning in the 1980s, many people were uneducated about condoms and reluctant to use them. People had a variety of legitimate and illegitimate fears about condoms such as believing that if a condom came off during sex, it might lodge in the womb and kill the woman.

Beginning in 1989, Uganda began receiving millions of condoms from abroad. In 1991 the media began to dispel myths about condoms and to encourage their use. Condom social marketing advertisements appeared in *The New Vision*, but created controversy and were then banned for several years. In an effort not to offend religious or other groups, the Uganda AIDS Commission pursued a policy of “quiet promotion”. However, the social marketing of condoms increased and condom shipments greatly increased. During the early to mid-1990s, faith communities that had initially opposed the promotion of condoms reduced their opposition when they continued to see so many people dying of AIDS. During the latter part of the 1990s there may have been more emphasis on condoms than on being faithful.^21^

## DISCUSSION

The collective evidence across these seven types of data supports the conclusion that, in response to the HIV/AIDS epidemic in Uganda, people in the late 1980s and early 1990s first restricted their sexual activity outside long-term marital and cohabiting relationships and then began using condoms if they had casual sexual partners. As a result, the incidence and prevalence of HIV decreased.

Of course, each of the seven types of evidence has important limitations for explaining the changes in sexual behaviour that led to the decrease in HIV incidence and prevalence in Uganda. However, to a remarkable extent, the different types of evidence are consistent with the conclusion that partner reduction and then condoms helped reduce the spread of HIV. This consistency adds greater credibility to the evidence as a whole.

In addition, the limitations of one method are sometimes offset by the strengths of another. For example, the newspaper accounts can accurately describe the timing of some events but cannot describe the representativeness of behaviour change. In contrast, the nationwide surveys were representative but cannot describe the exact timing of the beginning of behaviour change. Thus, collectively, these different methods provide multiple kinds of evidence for a consistent picture of behaviour change (table 1).

**Table 1:** Summary of evidence for changes in behaviour

	Strength of evidence	Conclusions
Modelling of HIV incidence and prevalence	Moderately strong for timing	Suggest incidence peaked about 1987/8 and began to decline
		Suggest incidence declined more rapidly about 1993
Reports of behaviour change in newspaper articles	Strong for timing; very weak for representativeness	Indicate behaviour change began in 1987 in Kampala and some other places in Uganda
		Suggest initial primary behaviour change was greater faithfulness (fewer casual partners) and not greater condom use
DHS and GPA surveys with questions about personal behaviour change	Strong for representativeness; weak for validity	Indicate large percentage decrease in sex before or outside marriage
		Indicate small percentage began using condoms
DHS survey data	Very strong for representativeness	Demonstrate small increase between 1988/9 and 1995 in all women who abstained from sex during the previous year
		Demonstrate large increase between 1988/9 and 1995 in young single women who abstained from sex during previous year
		Demonstrate large increase in condom use during sex with non-marital and non-cohabiting partners by 1995, especially in urban areas
GPA survey data	Moderately strong for representativeness	Among all women, suggest large decrease between 1989 and 1995 in percentage who had sex with non-marital or non-cohabiting partners
		Among married women, suggest very low and stable percentage who had extramarital sex
		Among young women, suggest large decrease between 1989 and 1995 in percentage who had premarital sex
		Among single women, suggest decrease between 1989 and 1995 in percentage who had two or more partners
		Among all women, demonstrate large increase in condom use during sex with non-marital and non-cohabiting partners by 1995, especially in urban areas
		Among single men, suggest large decrease between 1989 and 1995 in percentage who had premarital sex
		Among all men, suggest large decrease between 1989 and 1995 in percentage who had sex with non-marital or non-cohabiting partners
		Among married men, suggest large decrease between 1989 and 1995 in percentage who had extramarital sex
		Among young men, suggest decrease between 1989 and 1995 in percentage who had premarital sex
		Among single men, suggest decrease between 1989 and 1995 in percentage who had two or more partners
		Among all men, demonstrate large increase in condom use during sex with non-marital and non-cohabiting partners by 1995, especially in urban areas
Other surveys of sexual behaviour	Weak for representativeness	Suggest decrease in the number of sexual partners
		Suggest delays in the initiation of sex
		Suggest increase in the use of condoms
		Suggest moderately high level of condom use during casual sex in Kampala and other selected places by 1993
Reports of shipments of condoms to Uganda	Strong for timing; strong for validity of receipt; weak for actual condom use	Suggest relatively few condoms in Uganda before 1989
		Demonstrate that the number of condoms received in Uganda grew roughly exponentially
		Demonstrate that there were substantial numbers of condoms in Uganda by 1993
Historical documents describing programmatic efforts to address AIDS	Strong for timing; strong for validity	Demonstrate that, beginning about 1986, programmatic efforts focused primarily on being faithful and partner reduction
		Demonstrate that, beginning in the early 1990s, condom promotion and provision encouraged condom use

DHS, Demographic and Health Survey; GPA, the World Health Organization’s Global Program on AIDS.

The modelling indicates that the incidence peaked in about 1987 or shortly thereafter and declined more rapidly in about 1993. This suggests that behavioural changes had taken place by 1987 and continued at least through 1993. Newspaper articles show that the primary behavioural change began as early as 1987 and first involved less sex outside of marriage and not greater use of condoms.

Reports of perceptions of changes in personal sexual behaviour in the large national DHS and GPA surveys conducted in 1988/9 and 1995 provide stronger evidence that small percentages of people had begun to change their behaviour by 1988/9 and much larger percentages by 1995. They also indicate that much larger percentages of people restricted sexual activity than used condoms.

The DHS and GPA surveys provide even stronger data showing that, between 1988/9 and 1995, single men and women became less likely to have sex and began having sex with fewer sexual partners if they did have sex. These survey data also indicate that married men became less likely to have affairs and that both men and women began having sex with fewer casual partners.

The results of these nationwide surveys are consistent with other cross-sectional surveys and the survey assessments of personal behaviour change, all of which demonstrate that the most common behavioural changes involved reductions in sex outside marriage/cohabiting relationships and in numbers of sexual partners, rather than increases in condom use. This reduction in number of partners undoubtedly included a reduction in concurrent sexual partners as well as sequential partners. This is particularly important because the rapid rise in HIV rates in Uganda, especially in Kampala, suggests that HIV may have spread especially rapidly through sexual networks of concurrent partners as well as more slowly through sequential partners.^4^

**Key messages**

- Multiple sources of evidence, including models of the prevalence and incidence of HIV, newspaper reports, surveys, shipments of condoms and HIV prevention activities, provide a consistent picture of when sexual behaviours changed and which sexual behaviours changed.
- First, people restricted their sexual activity by being less likely to have sex if single, being less likely to have extramarital affairs if married, and having fewer casual sexual partners. Then, those people still having casual sex increased their use of condoms with non-marital/non-cohabiting partners.
- Breaking up the sexual networks by having fewer sexual partners and then decreasing the risk of STD transmission through the use of condoms is a powerful combination that markedly reduced HIV transmission.

The DHS, GPA and other surveys also indicated that few people used condoms before 1989 but that condom use increased, especially in urban areas and especially with casual sexual partners. These survey data are consistent with the very large increases in the shipments of condoms received in Uganda in the early 1990s, shipments that demonstrate the availability and presumably the need for condoms.

In numerous qualitative analyses, many focus group and interview respondents indicated that first there was a change in norms regarding casual sex and a reduction in casual sex, followed by greater acceptance of condoms and condom use.

Finally, the temporal order of these behaviour changes is also supported by programmatic efforts of the times. First the emphasis was on “zero grazing” and “being faithful” then, beginning in 1991, the emphasis on condom use increased markedly. This combination—breaking up the sexual networks by having fewer sexual partners and then decreasing the risk of STD transmission through the use of condoms—is a powerful one. Modelling of the relationship between number of sexual partners and the size of sexual networks demonstrates that, in general, even small decreases in the mean number of sexual partners can sometimes markedly reduce the size of sexual networks.^37^ The reductions in numbers of sexual partners in Uganda demonstrated by multiple kinds of data certainly had the potential to break up these sexual networks and thereby reduce the transmission of HIV. The greater use of condoms in the remaining smaller sexual networks further reduced HIV transmission. Then, according to multiple sources, the incidence and prevalence of HIV declined dramatically. These findings are consistent with other analyses of declines in HIV prevalence in other generalised epidemics in sub-Saharan Africa (eg, in Kenya and Zimbabwe), which suggest that giving a strong emphasis to partner reduction while also encouraging condom use (and abstinence) is much more effective than primarily promoting condom use (or abstinence).^38^ ^39^
